# *In Silico* Investigation of Novel Compounds as Inhibitors of Acetylcholinesterase Enzyme for the Treatment of Alzheimer's Diseases

**DOI:** 10.1155/2024/2988685

**Published:** 2024-02-08

**Authors:** Kassim Adebambo, Oghenekevwe (Claudia) Ojoh

**Affiliations:** Department of Clinical Pharmaceutical and Biological Science, University of Hertfordshire, Hatfield, UK

## Abstract

Alzheimer's disease (AD) is a “progressive, neurodegenerative disease that occurs when nerve cells in the brain die.” There are only 4 drugs approved by the United States Food and Drug Administration (FDA). Three (donepezil, rivastigmine, and galantamine) out of these four drugs are anticholinesterase inhibitors, while the fourth one memantine is an N-methyl-D-aspartate (NMDA) receptor inhibitor. Currently, two immunotherapy drugs that target amyloid protein (donanemab and lecanemab) are being considered for the treatment of Alzheimer's disease at an early stage. All these drug molecules are still not the complete answer to the treatment of Alzheimer's disease. A recent report from the Office of National Statistics showed that AD is the leading cause of death in 2022. Therefore, there is an urgency to develop more drugs that can treat AD. Based on this urgency, we aim to investigate how bioactive and already approved drugs could be repurposed for inhibiting the anticholinesterase enzyme using computational studies. To achieve this, the data science tool—Python coding was compiled on Jupyter Notebook to mine bioactive compounds from the ChEMBL database. The most bioactive compounds obtained were further investigated using Molecular Operating Environment (MOE) software to carry out molecular docking and ligand analysis, and this was followed by molecular dynamics simulation production at 35 ns using GROMACS 2022.4 on Archer 2 machine. The molecular dynamic analysis was carried out using HeroMDanalysis software. Data mining of the ChEMBL database was carried out for lipase inhibitors, and this gave CHEMBL-ID 1240685, a peptide molecule, the most active compound at the time of data mining. Further literature studies gave Zoladex an FDA-approved drug for the treatment of breast cancer as another compound of interest. The *in silico* studies were carried out against the anticholinesterase enzyme using two FDA-approved drugs donepezil and galantamine as a template for comparing the *in silico* activities of the repurposed drugs. A very useful receptor for this study was PDB-1DX6, a cocrystallized galantamine inhibitor of acetylcholinesterase enzyme. The molecular docking analysis (using ligand interactions) and molecular dynamic analysis (root mean square deviation (RMSD) and root mean square fluctuation (RMSF)) showed that the two peptide molecules CHEMBL-1240685 and Zoladex gave the best binding energy and stability when compared to the FDA-approved drugs (donepezil and galantamine). Finally, further literature studies revealed that Zoladex affects memory reduction; therefore, it was dropped as a possible repurposed drug. Our research showed that CHEMBL-1240685 is a potential compound that could be investigated for the inhibition of anticholinesterase enzyme and might be another drug molecule that could be used to treat Alzheimer's disease.

## 1. Introduction

Alzheimer's disease (AD) is a “progressive, neurodegenerative disease that occurs when nerve cells in the brain die” [1]. It is the most common form of dementia [2] and affects more than 25 million people [3]. AD is an important disease to address as it leads to prolonged suffering and death for many individuals and families—it is the sixth highest cause of death in the United States, and nearly 4% of deaths in the country were caused by it [2].

Symptoms of AD include a deficit in memory and gradual memory loss. This includes deficits in working memory, which is the capability of maintaining and manipulating information required to be retained short term [4], and the gradual deterioration of the more clinically relevant type of memory affected in AD patients; Alzheimer's disease can result in episodic memory, which makes it difficult for a person to consciously retrieve previous experience or various past episode in the life of AD patient [4, 5]. Additional AD symptoms include behaviour (changes in behaviour), such as depression [6] and delusions. The most common behavioural symptom manifests in a strong but false mental belief held by AD patients [7, 8].

The prevalent demography of Alzheimer's disease (AD) includes age, down syndrome patients, and women, with age being the greatest risk factor for developing AD [9]. This is because the risk of developing AD increases with age, and it is most common in people aged over 65 years. AD is estimated to affect 1 in 14 people aged over 65 years old and 1 in 6 people over 80 years of age [10]. AD is an important disease to address, according to Alzheimer's Research UK release of 12^th^ April 2023, and the Office of National Statistics has revealed that AD was the leading cause of death in 2022.

A significant amount of research that has been carried out in attempts to treat Alzheimer's disease (AD) utilises the “Amyloid Hypothesis” as the main explanation for the cause of AD. The amyloid hypothesis specifies that the primary cause of Alzheimer's disease is the accumulation and deposition of oligomeric amyloid beta (A*β*) peptide in the brain [11]. However, research conducted by Kim et al. [12] and Kim et al. [13] suggested otherwise.

Kim et al.'s [13] research shows that, despite A*β* amyloid fibrils, amyloid deposits, and A*β* oligomers being present in BRI2-A*β* mice due to the overexpression of amyloid beta 42 (A*β*42), the deterioration of nerve cells and the loss of neurons did not occur, which would be expected in AD patients when assuming the amyloid hypothesis as the cause of AD. Furthermore, additional research by Edison et al. [14] and Li et al. [15] found that neurotypical patients were with amyloid deposits and that, even more intriguingly, there were AD patients with hardly any amyloid deposits, which is unexpected for AD patients. These studies show that neurodegeneration and amyloid deposition are independent of each other, which contradicts the amyloid hypothesis.

The findings discussed above also show that A*β* is not naturally cytotoxic and, so, puts the amyloid hypothesis into question regarding its validity for being the primary explanation and cause of AD. Furthermore, these findings also support the fact that more hypotheses regarding the cause of AD need to be thoroughly investigated for advances in AD treatment to exist more promptly and that focusing on a singular hypothesis as the cause of AD could significantly hinder the rate at which novel drug candidates for AD are being discovered. This makes sense as there are currently only 4 drugs that are approved by the United States Food and Drug Administration (FDA) for the treatment of AD [16] since the first diagnosis of AD was given [17], which could be due to this focusing on the amyloid hypothesis. Investigating more hypotheses regarding the cause of AD could render AD's treatment more effective and lead to a breakthrough in the discovery of novel drugs for treating the disease.

Furthermore, assuming the amyloid hypothesis as the primary explanation of AD deprives other plausible explanations for being the cause of AD, such as the involvement of glycogen synthase kinase 3 *β* (GSK-3*β*) in the pathogenesis of AD. Research shows that increased GSK-3beta activity is linked to increased A*β* production and A*β* deposits, along with the hyperphosphorylation of the protein [18, 19], Tau, which is observed in AD. There is also the GSK-3*β* hypothesis which states that it is due to the “over-activity of GSK3*β* accounts for memory impairment, tau hyper-phosphorylation, increased *β*-amyloid production and local plaque-associated microglial-mediated inflammatory responses” observed in AD [12, 20–22].

From the initial discussion above, we have mentioned that there are currently only four small-molecule drugs that have been approved by the United States Food and Drug Administration (FDA) for the treatment of AD since the first diagnosis of AD. Meanwhile, these four types of small drug molecules were approved to temporarily treat the symptoms of Alzheimer's disease thereby slowing down the rate at which Alzheimer's progresses in patients—out of these four drugs, three act by inhibiting the phase of acetylcholinesterase enzyme, and they are known as acetylcholinesterase enzyme inhibitors (AChEi), and these are donepezil, rivastigmine, and galantamine [23]. The AChEi drugs work by preventing the breakdown of acetylcholine in the brain, because the breakdown of acetylcholine in the brain distorts the communication between the nerve cells in the brain. The fourth drug currently in use is memantine which is an antagonist of the N-methyl-D-aspartate (NMDA) receptor. Newcomer et al. [24], in a review, revealed that a high level of the NMDA receptor in the brain is associated with memory impairment, and their report also shows that at old age, the NMDA attains a hypofunctional level which contributes to memory loss at an old age. Therefore, memantine inhibits the effect of the hypofunctional level of NMDA.

### 1.1. Importance of Acetylcholine in Memory

Acetylcholine is a very important compound that is very useful in the transmission of information to the brain. Research by Fioravanti and Yanagi in 2005 [25] showed that the administration of CDP-choline to elderly people has a positive effect on the memory function of the elderly people. Further studies by Zielsel et al. [26] showed that CDP-choline repaired the cell membrane and enhanced the synthesis of acetylcholine in the membrane. Saver [27] gave a comprehensive review of how choline can facilitate the synthesis of phosphatidylcholine, and in this review, acetylcholine was shown to be involved in the quick repair of mitochondrial membranes. Acetylcholine (ACh) is present at the interneuron pathways in the brain which enhances information flow to the brain. The loss of this compound is associated with memory loss which results in Alzheimer's disease [28].

### 1.2. Ache and Its Involvement in Alzheimer's Disease

The proper function of the brain has been linked to the acetylcholine activities in the brain [29]; therefore, an increase in the acetylcholine in the neurotransmission process will improve the memory of a patient, although Madav et al. [30] have described various attempts that were investigated by other researchers which targeted amyloid *β* accumulation, and tau protein hyperphosphorylation, and these investigations have not yet resulted in any FDA-approved drug for the treatment of Alzheimer's disease. Recently, two drug molecules have been celebrated as a game changer in the treatment of Alzheimer's disease. The celebrated drugs are donanemab and Lecanemab; both drugs are classed as immunotherapy drugs, and they target the amyloid protein. According to Sara Readson in the Nature report of 27^th^ July 2023, donanemab, when taken at the earlier stage of dementia, led to about 47% of the patients having no Alzheimer's disease progression after one year [31]. Lecanemab has already been approved in the USA for the treatment of early-onset dementia. While these two drugs are landmark breakthroughs in the treatment of dementia, more work is still needed in the development of new drugs for the treatment of Alzheimer's at any stage.

Apart from these two immunotherapy drugs being developed, the only available drugs are those that control the activity of the acetylcholinesterase enzyme, thereby enhancing the effect of cholinergic neurotransmission activities and ultimately improving the memory function of the brain. Acetylcholinesterase enzyme functions by hydrolyzing the serine found in the cholinergic synapses of the brain. This resulted in the breaking down of acetylcholine into acetate and choline, thereby terminating the neurotransmission activities of acetylcholine in the cholinergic system and leading to memory loss [32]. Inhibiting the action of acetylcholinesterase enzyme has been a good target for the development of Alzheimer's drugs, and this is the major focus of this research work.

Donepezil is used to treat the mild to moderate symptoms of AD where it functions by reversibly inhibiting the cholinergic enzyme, acetylcholinesterase (AChE). Inhibiting AChE prevents it from lysing the neurotransmitter, acetylcholine, thus, allowing the increase of acetylcholine levels in neuromuscular junctions in the brain to occur, thereby, helping to prevent the loss of the cholinergic neurons' function that is experienced in AD patients [33].

Rivastigmine and galantamine function similarly to donepezil, by inhibiting acetylcholinesterase (AChE), except that galantamine is also a competitive, reversible, and specific inhibitor of AChE.

Based on the dangerous effects of Alzheimer's disease and the fact that the drugs that have been approved only address patients at an early stage of Alzheimer's disease onset, it is paramount to investigate more drugs for the treatment of Alzheimer's disease. Furthermore, bringing a new drug to market is capital-intensive, and it takes an average of 17 years. It is very important to investigate if the already approved drugs and some drugs that have been bioassayed could be repurposed for the treatment of Alzheimer's disease. Therefore, this research is aimed at using machine-generated data to repurpose FDA-approved drugs and new bioactive molecules from the ChEMBL database from their original biological application for the inhibition of anticholinesterase enzyme which has been shown from our discussion above to be a biological target for the treatment of Alzheimer's disease. The drug repurposing research was set to investigate if the new compound mined from the ChEMBL database using Python coding could be repurposed to treat Alzheimer's disease, and the *in silico* repurposing experiment will be based on molecular docking and molecular dynamics simulation. The virtual control that was employed for this experiment was the FDA-approved drugs (donepezil and galantamine).

## 2. Methodology

This research work is based on using data science tools (Python on Jupyter Notebook) to obtain bioactive compounds from the ChEMBL database. The first database that we screened was bioactive compounds against lipase enzyme and investigated how the best molecule could be repurposed for the treatment of other diseases, and in this case, we investigated the use of the bioactive compounds for the inhibition of acetylcholinesterase enzyme in the treatment of Alzheimer's disease. The activity of the bioactive compound was investigated *in silico* using molecular docking and molecular dynamics simulations. To achieve our aim of repurposing drugs, two AChEi drugs already approved for the treatment of Alzheimer's disease—donepezil and galantamine—were used as the template for comparing the binding activity and stability of the repurposed drug. The receptor of choice was PDB 1DX6 because it is the crystal structure of the anticholinesterase activity of galantamine, with a resolution of 2.3 Å.

### 2.1. Data Mining of ChEMBL Database

The diversity of different bioactive compounds deposited in the ChEMBL database has made the database a very good source for data mining [34], and to facilitate the development of drugs using Python, CHEMBL created a client library that enables researchers to develop python codes for mining the various data on the ChEMBL library [35].

Through the use of the web-based interactive computing platform Jupyter Notebook, accessed from the Anaconda Python Package Manager, it was possible to construct a code that could collect compounds from the ChEMBL database. The ChEMBL database amasses over 2.3 million bioactive compounds with drug-like properties.

As stated in the preamble to the methodology, writing codes are aimed at filtering through the ChEMBL database to discover bioactivities of already assayed compounds for any diseases and repurpose the bioactive compounds. In this research, Python search of the ChEMBL database was for compounds that have been assayed as lipase inhibitors towards the development of new drug candidates for the treatment of pancreatic cancer, and a detailed report on lipase inhibitors can be described in a review by Kumar and Chauhan [36]. Lipase inhibitors were used as the search term in the Python code. The search returned 41 molecules. Python code was developed to remove duplicates which resulted in 37 structures. Further code was composed to obtain the IC50 of these bioactive molecules and sort them out according to the decrease in bioactivity.

### 2.2. Protein Preparation

The crystal structure of the protein used in this study was PDB-1DX6, this crystal structure is the structure of acetylcholinesterase complexed with (-)-galantamine at 2.3 Å, and the structure was developed by Greenblatt et al. [37]. 2.3 Å is still a good resolution that shows the compactness of the protein structure, according to the guide to understanding PDB (PDB-101: Learn: Guide to Understanding PDB Data: Resolution (http://rcsb.org/)). PDB crystal between 1 Å and 2.99 Å can be said to be of high-quality structure, while any PDB structure over 3 Å or higher has low quality.

The Molecular Operating Environment (MOE) software developed by Chemical Computing Group (version 2020.0901) was used to prepare proteins and ligands for molecular docking study. The protein preparation was achieved by loading 1DX6 onto to MOE panel. Sequence analysis was performed on the 1DX6 protein to analyse its amino acid sequences and determine if there is any missing link in the sequence. The “Quick prep” command in the MOE panel was used to correct the anomalies in the protein by protonating the structure, refining the root mean square deviation (RMSD), performing energy minimization, and making it ready for docking. Site finder command on the MOE panel gave the binding pocket of the cocrystallized ligand in the receptor, and the binding pocket is the region within the protein that possesses suitable properties for binding a ligand [38].

### 2.3. Ligand Preparation

The ligand preparation was carried out by using SMILES of donepezil, galantamine, and Zoladex obtained from PubChem while the SMILES of CHEMBL-1240685 was obtained from the ChEMBL database on 10/11/2022. The 3D structures of these compounds were created by using the builder command of the MOE software, the 3D structure was prepared and minimised on the MOE software, and the ligand preparation added hydrogen atoms and ionized it at pH; energy minimisation is necessary to reduce the strain within the bond and relax the molecule for a biological system.

### 2.4. Molecular Docking

Molecular docking is an automatic procedure that docks drug molecules into the binding pockets of a given receptor [39]. This process is aimed at predicting the structure and stability of the ligand-receptor complex formed by the ligand and receptor that are interacting together. This interaction can automatically reveal the types of cellular responses that might be taking place when the drug molecules enter the biological system [40]. The molecular docking was executed by a search algorithm that continually analyse the conformation of the ligand until the minimum energy of the ligand has been reached [41]. The molecular docking algorithm also quantitively predicts the binding affinity, the number, and the types of interactions that ligands will have with the protein's binding site [39]. The docking was carried out using the dock command on the MOE software, the docking protocol involved generating 30 different poses, and the system selected the best five docking scores for analysis.

### 2.5. Molecular Dynamics Simulation

The molecular dynamics simulation was conducted on the GROMACS 2022.4 version, the instruction in the GROMACS tutorial for protein-ligand interactions by Lemkul [42] was followed step by step, and *CHARMM-36 all field* was selected both for protein topology generation and ligand topology generation. Another amendment that was made to the GROMACS tutorial was in the molecular dynamics simulation steps which was used in the mdp file of the molecular dynamics production run. The number of steps used was 17500000 (equivalent to 35000 picoseconds (35 ns) with a timestep of 2 fs (2 femtoseconds)). The trajectory file (.xtc) generated was analysed using the HeroMDanalysis program and Xmgrace. For HeroMDanalysis, the simulation files used are the tpr, .xtc, and .edr files [43]. Finally, 2D snapshots of the molecular dynamics simulation steps were taken at 10 ns and 35 ns to check the types of contacts that were made during the molecular dynamics simulation.

### 2.6. *In Silico* Absorption, Distribution, Metabolism, Excretion, and Toxicity (ADMET) Studies

Parmar et al. [44] have described how *in silico* data could be used to predict the ADMET of potential biologically active compounds. The only amendment to their method was that the SwissADME online tool was used to study the ADME of the compounds. The toxicity data were obtained using ProTox-II based on the report of Shah et al. [45]. Following the *in silico* toxicity experiment of Shah et al. [45], the following toxicity data were also collated in this research: hepatoxicity, carcinogenicity, mutagenicity, cytotoxicity, and toxicity level.

### 2.7. *In Silico* Bioactivity Analysis

*In silico* bioactivity data were also obtained for all four compounds investigated using the tool Molinspiration Cheminformatics server (http://www.molinspiration.com). As reported by Shah et al. [46], this technique made use of a Bayesian statistical model to carry out *in silico* bioactivity prediction of potential drug candidates.

## 3. Results and Discussion

This research is aimed at repurposing bioactive compounds. Drug development for the treatment of a disease takes a long time, because a new drug candidate, apart from its medical use, must be safe to use by patients. It has been suggested that to bring a new drug to market, it takes around 17 years and almost $2 billion [47]. Therefore, a new approach is needed towards discovering new drug entities for treating diseases, and this led to drug repurposing [48, 49]. Low et al. [50] in their review suggested that drug repurposing can save pharmaceutical companies up to 300 million dollars. Therefore, in our approach to contribute to drug retargeting or repurposing, we used data science tools—Python coding—to search the ChEMBL database for some bioactive compounds starting from bioactive compounds that have been deposited in the ChEMBL database as lipase inhibitors. The coding search gave CHEMBL-1240685 as the most active molecule with IC50 of 1.6*E* + 4 nM. The CHEMBL-1240685 was further investigated alongside Zoladex—a brand of Goserelin that has been approved for the treatment of breast cancer patients (Cancer Research UK [51]). Galantamine and donepezil were used as the template for comparing the *in silico* activity of the two drugs; we were hoping to repurpose because they have already been approved by the FDA as drugs that can be used to slow down the progression of Alzheimer's disease, and they both target anticholinesterase enzyme.

### 3.1. Molecular Docking

**Figure 1:**
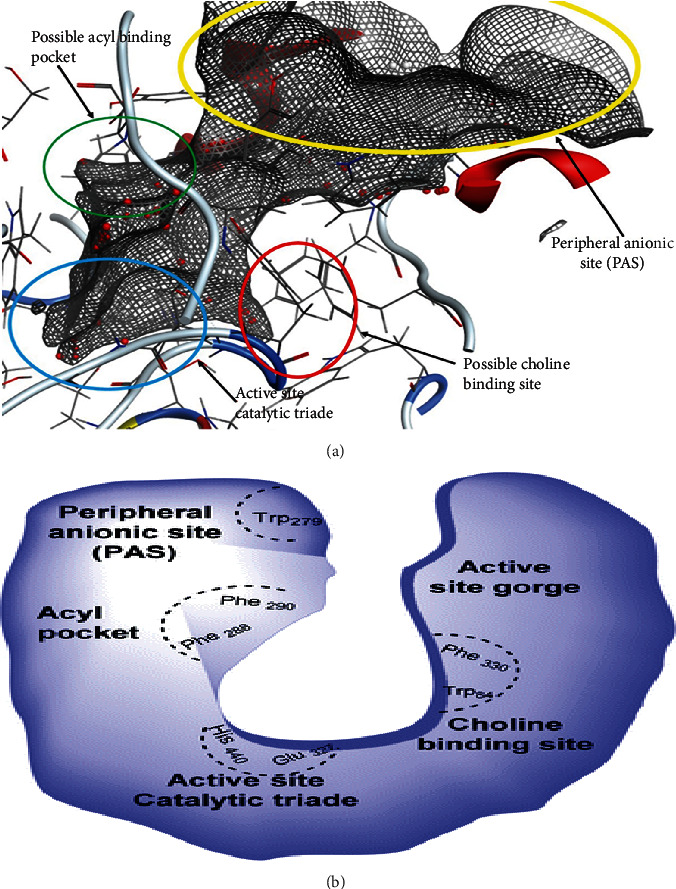
(a) Vander waal surface of the receptor showing the binding pocket of 1DX6 created by us on the MOE software. (b) Visual representation of the binding pocket as shown by Kareem et al. [53] and also supported by Colovic et al. [32].

Molecular docking is an *in silico* modelling approach that enables us to understand the interaction between protein and ligands, and this approach fits ligand into the identified binding pocket of the protein; also, the types of interactions within the binding pocket show whether the interactions are hydrophilic or hydrophobic. The poses generated were ranked according to their binding affinity during the protein-ligand interactions. According to Bhardwaj et al. [52], molecular docking experiment enables us to understand whether the protein-ligand will lead to a favourable binding affinity or not. In this research, the receptor used for the study was the PDB-1DX6 (structure of acetylcholinesterase complexed with (-)-galantamine). The binding pocket of the receptor was identified by using the MOE software. This reveals that the binding pocket of the receptor is the same as that of the cocrystallise ligand (galantamine) (Figure 1).

**Figure 2:**
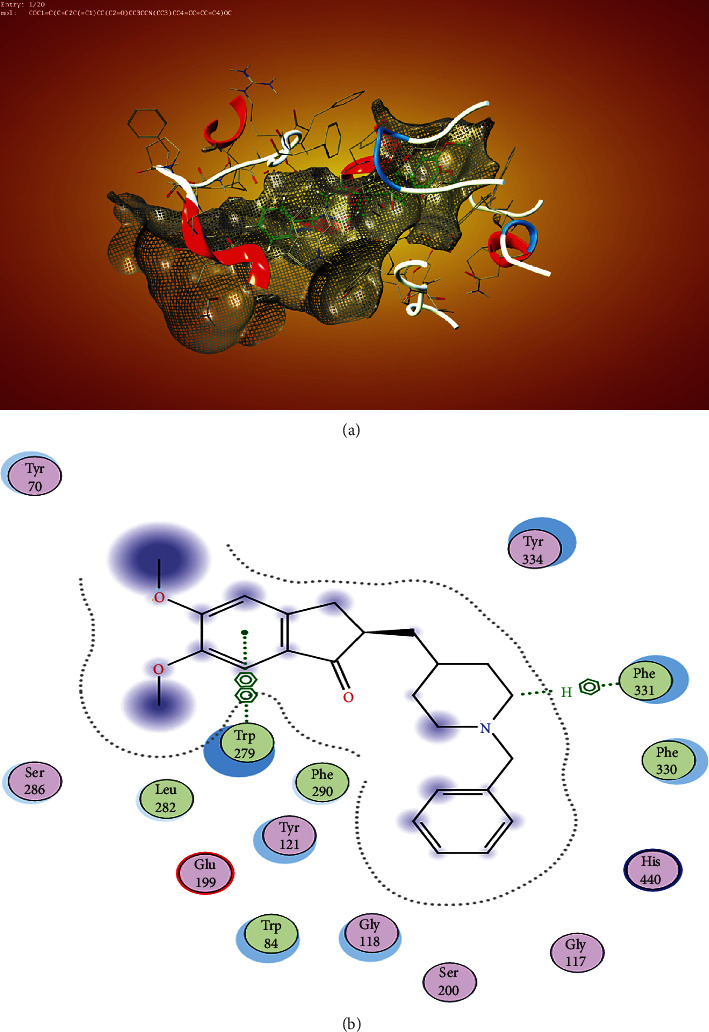
(a) Surface mapping of donepezil in the binding pocket and none of the groups on the ring passing through the surface, which shows that there was favourable contact with the binding pocket. (b) The ligand interaction was with 6-ring PHE 331 and 6-ring TRP 279.

Acetylcholine esterase binding pocket can be more visualised as shown in Figure 1(b), and according to Kareem et al. [53] and Colovic et al. [32], the enzyme has four sites for drug interaction—these are the active site (catalytic triade), the peripheral anionic site, acyl pocket, and the choline binding site. The amino acids in these sites are histamine (His), serine (Ser), and glutamine (Glu) for the catalytic site while the peripheral site has TRP and Asp; in the choline site, there are two PHE and TRP amino acids and, finally, in the acyl pocket, two PHE amino acids. The idea of these two authors (Rzgar Tawfeeq [32, 53]) was used to give possible labelling of the various position in the binding pocket. The binding interactions of each of the ligands investigated are presented (Figure 2).

Figure 2(a) revealed the position of donepezil in the binding pocket, and it was observed that the ligand was not experiencing any clashing with the binding pocket because none of its carbon chain protruded out of the pocket.  Figure 2(b) shows clearly that donepezil has two interactions with the amino acids in the binding pocket—one interaction was with the amino acid in the area of the choline binding pocket (PHE 331), and the second interaction was with the amino acid around the peripheral anionic site of the protein (TRP 279).

**Figure 3:**
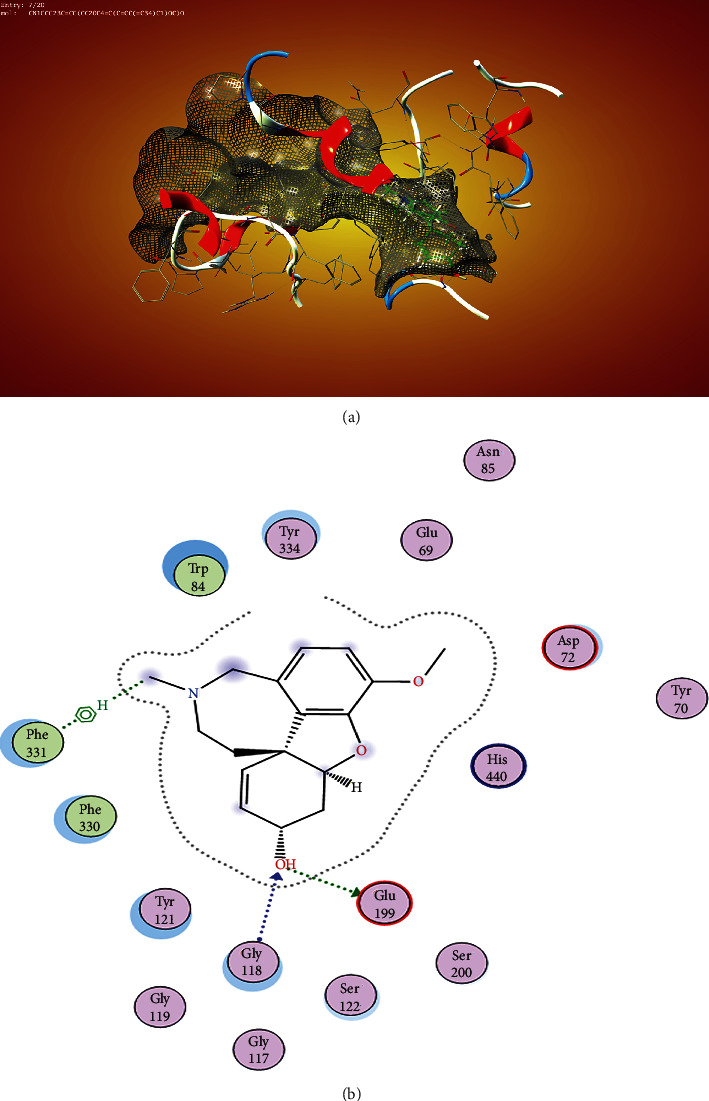
(a, b) Galantamine in the binding pocket. It occupies only the enzyme catalytic site. The ligand interactions are with GLU 199, GLY 118, and 6-ring PHE 331. (a) There was favourable contact with the binding pocket.

Furthermore, the cocrystallise ligand galantamine had three ligand interactions within the binding pocket (Figure 3(b)); the ligand interactions are all within the region of the enzyme catalytic site, and these are with amino acids PHE 331, GLU 199, and GLY 118.

The two FDA-approved ligands (donepezil and galantamine) showed favourable contact with the binding pocket, and the interactions were then used as a template to discuss the impact of the repurposed drugs in this study on the anticholinesterase enzyme.

**Figure 4:**
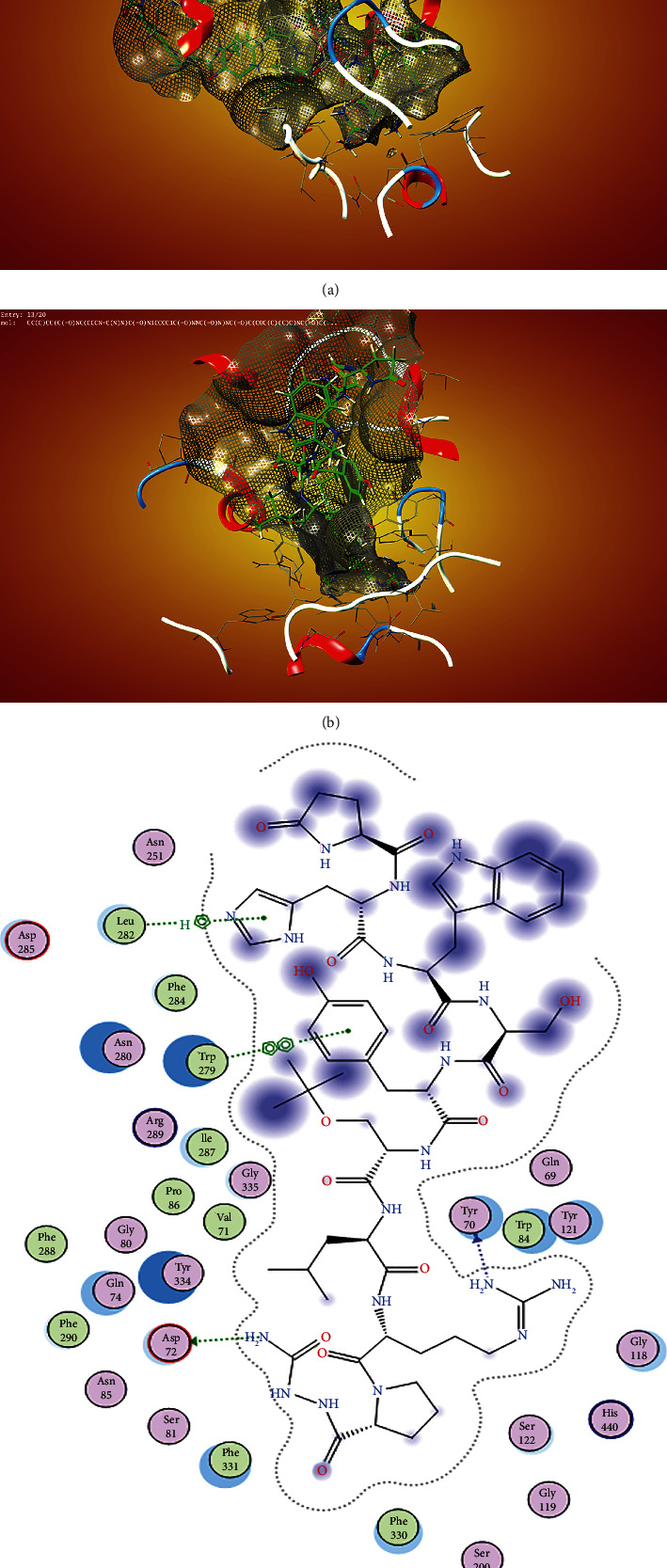
Interaction of Zoladex within the binding site of AChE shows interaction with the amino acids in the choline binding site TYR-70. Asp 72 in the acyl binding site and pi-pi interaction with the TRP-279 and LEU 282 around the peripheral anionic binding site.

Zoladex showed the four interactions within the binding pocket (Figure 4(c)) within the binding pocket of the anticholinesterase enzyme. This molecule perfectly occupied all the space within the binding pocket (Figure 4(b)), and the contacts were favourable as no substituent was sticking out of the surface mesh (Figure 4(a)).

**Figure 5:**
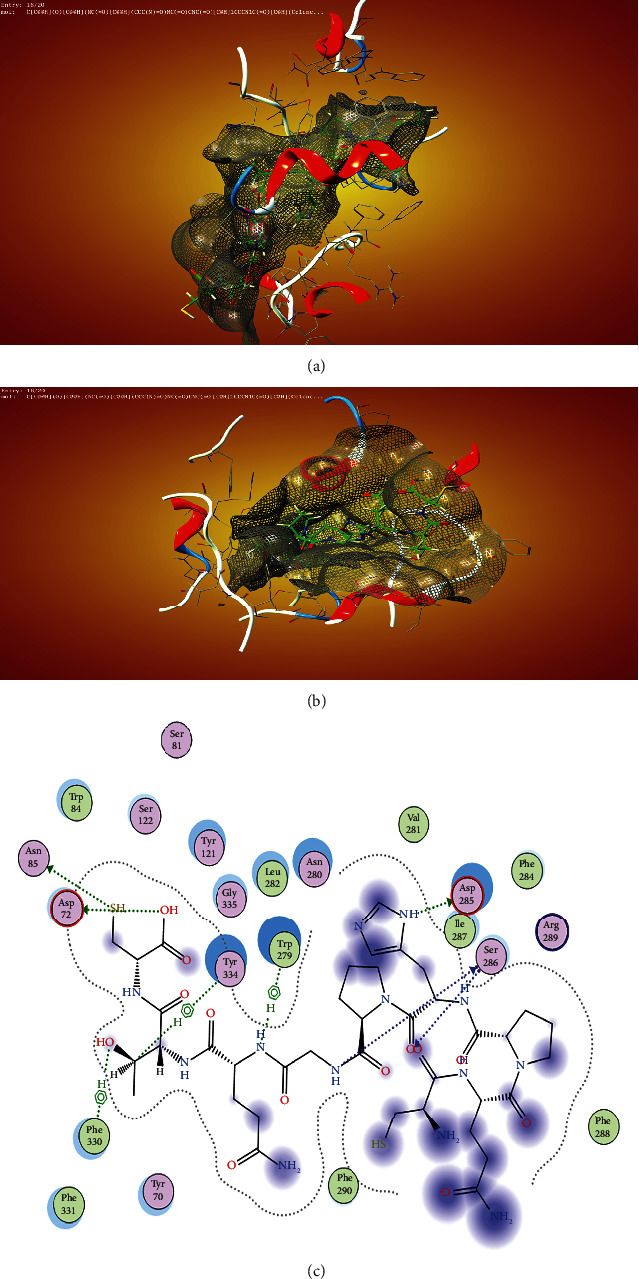
Ligand interaction of CHEMBL1240685 in the binding pocket of ACHE. (a) Ligand occupied the whole binding pocket, and the interaction was favourable. (b) The ligand made interactions with ASP72 and ASN 85 in the catalytic site region, ligand interactions with PHE 330 around the choline binding site region, and ligand interactions with TYR-334 and TRP 279 possibly around the acyl pocket. Finally, with SER 286 and ASP 285 around the peripheral anionic site.

Finally, the ligand interaction of CHEMBL-1240685 (Figure 5) showed that the molecule has a total of eight interactions with the receptor, and these interactions are SER 28, ASP 285 in the acyl binding pocket, ASN-85, TYR-334, and ASP-72 around the catalytic binding pocket; there was an interaction with the amino acid PHE-330 around the choline binding pocket and TRP-279 around the peripheral anionic site.

A summary of the molecular docking interactions is shown in Table 1, and from the biding affinity, CHEMBL-1240685 has the best ligand interaction and a very high binding affinity (-12.1467) in the MOE algorithm of docking scoring; the higher the negative value, the better the binding affinity. This was followed by Zoladex (binding affinity of -11.2118).

In Table 1, all the ligands have interactions within the binding pocket of the receptor, and CHEMBL-1240685 also showed a remarkably high binding affinity to the receptor when compared with all the ligands investigated.

### 3.2. *In Silic*o ADME Studies

SwissADME free online tools allow the ADME of the investigated compounds to be calculated. From Table 2, the selected compound CHEMBL-1240685 has a LogS similar to donepezil. The low GI permeability of CHEMBL-1240685 and Zoladex was not unexpected because of their large size, but the advances in drug delivery technology could easily be employed to overcome the effect of the low GI permeability of these large molecules. The usefulness of *in silico* data of compounds for predicting the ADME properties of possible hit compounds has also been used to predict the bioactivities of plant extracts as potential inhibitors of SARS-CoV-2 [44].

### 3.3. *In Silico* Toxicity Studies

The results of *in silico* toxicity studies conducted using ProTox-II are given in Table 3.

The *in silico* toxicity results showed that all four compounds have toxicity values between 4 and 5 except galantamine which has toxicity values of 3. This shows that the compound of choice CHEMBL-1240685 is not as toxic as other FDA-approved drugs with a toxicity level of 5 and LD_50_.

### 3.4. Molecular Dynamics Simulation

Molecular docking as shown above has been able to show the type of ligand interactions taking place within the binding pocket of the receptor (1DX6), and there are shortcomings in relying solely on molecular docking results to completely carry out our virtual screening of possible drug candidates. This shortcoming is a result of the fact that molecular docking did not show the atomic motion within the protein-ligand complex [54]. Molecular dynamics simulation can account for the calculation involving motion within the protein-ligand interaction [55]. Molecular dynamics simulation is based on the Newtonian calculation [56]. Furthermore, molecular dynamics simulation helps computational medicinal chemists to understand timely motion within a biological system. The use of molecular dynamics simulation has been argued to reduce the amount of time that could have been spent in the wet lab to determine the possibility of biological activity of new drug candidates [57].

To understand the stability and fluctuation of the protein-ligand interaction, molecular dynamics simulation was conducted for 35 ns on the Archer 2 machine. Root mean square deviation analysis (RMSD) was calculated for the complex. Furthermore, the fluctuation of the residue during the simulation was evaluated by calculating the root mean square fluctuation (RSMF) of the protein over the 35 ns period of simulation. The RMSF analysis reveals the flexibility of the protein within the active site [58].

**Figure 6:**
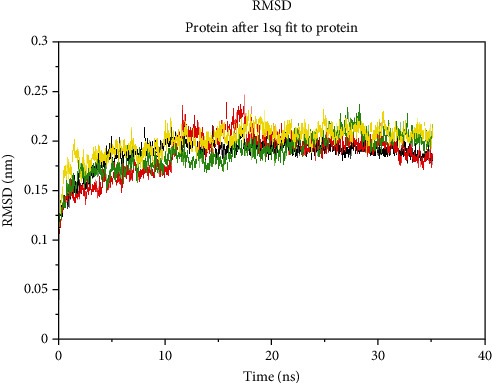
The protein's behaviour over the protein range: ligand interaction donepezil = black, galantamine = red, Zoladex = green, and CHEMBL-1240685 = yellow.

The RMSD analysis of the protein-ligand interaction of the four ligands (Figure 6) showed that the ligand interaction led to an RMSD variation within 0.1 nm to 0.2 nm, although around 12 ns to 20 ns, the RMSD of galantamine was higher than the remaining three compounds. Apart from this slight variation, the protein tends to be stable throughout the 35 ns simulation.

**Figure 7:**
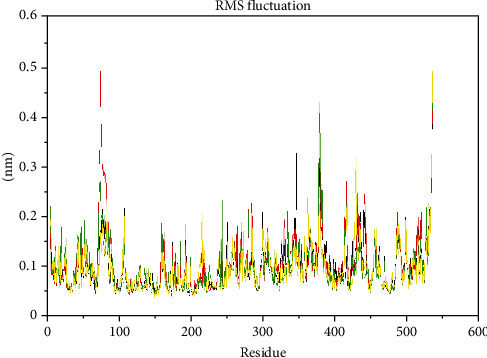
RMSF of the protein during the protein-ligand interaction simulation: donepezil = black, galantamine = red, Zoladex = green, and CHEMBL-1240685 = yellow.

The RMSF plot for the complex (Figure 7) showed that the residue fluctuation in the protein—galantamine complex—appeared to be more disturbed than the remaining three complexes around 100 residues. The higher fluctuation observed for the protein-Zoladex complex occurred around 360 residues. The fluctuation protein-ligand complex of donepezil and CHEMBL-1240685 is within the region of 0.1 nm to 0.25 nm. This shows that CHEMBL-1240685 and donepezil appear to have less flexibility within the binding pocket. According to De Vita et al. [59], a high RMSF value reveals that the protein was not making strong interactions with the ligand during the simulation, and their residue was not making maximum binding interactions with the ligands.

**Figure 8:**
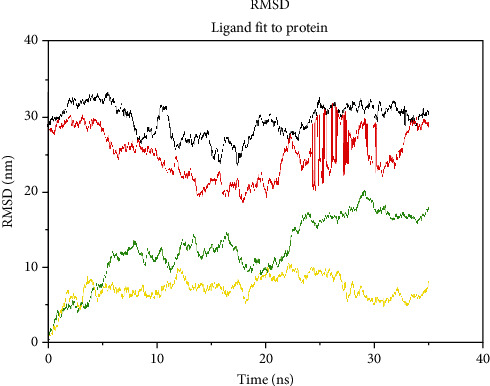
The behaviour of the ligands within the system during the 35 ns simulation: donepezil = black, galantamine = red, Zoladex = green, and CHEMBL-1240685 = yellow.

Further investigation of molecular dynamics simulation involved the analysis of how the ligand behaves within the binding pocket (Figure 8).

The root mean square deviation of the ligand within the binding pocket (Figure 8) revealed that out of all the four ligands, CHEMBL-1240685 has the least RMSD value and hence greater binding stability with the receptor, and this also confirms the high binding affinity of the ligand observed during the molecular docking analysis of the ligand. From the *in silico* experiment, we can infer that CHEMBL-1240685 is a possible hit compound that can be investigated as a new anticholinesterase enzyme inhibitor compound.

**Figure 9:**
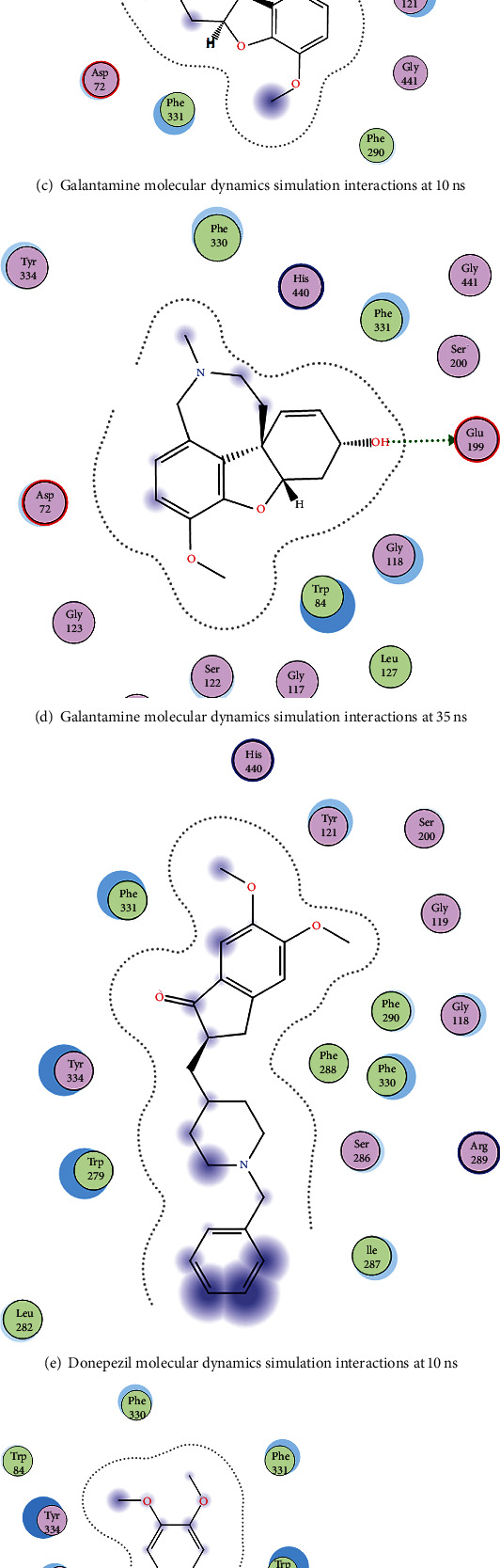
(a–h) 2D diagram of contact points during the molecular dynamics simulations as obtained from the ligand interaction analysis of the snapshot taken at 10 ns and 35 ns timestep using the MOE software to study the ligand interactions of the snapshot.

Molecular dynamics enables us to understand the stability of the protein-ligand interactions, and in addition to the RMSD, and RMSF data shown in Figures 6–8 and a 2D snapshot of the ligands (Figures 9(a)–9(h)) revealed the types of protein-ligand interactions that were taken place during the molecular dynamics simulations. These snapshots were taken at 10 ns and 35 ns. This shows that the large molecules Zoladex and CHEMBL-1240685 made contact at 10 ns and at the end of the simulation (35 ns), whereas the small molecules took a longer simulation time before interacting with the receptors. The molecular dynamics simulation gave good information about the pharmacodynamics and pharmacokinetics of the drug molecules [60].

## 4. Conclusion

The binding analysis and molecular dynamics simulation have shown that CHEMBL-1240685 which has been assayed as an inhibitor of lipase enzyme in the treatment of pancreatic cancer could be investigated for possible repurposing in the treatment of Alzheimer's disease, and our study has shown that CHEMBL-1240685 has a greater binding affinity, ligand interactions, and stability in the binding pocket of ACHE.

According to the data obtained, CHEMBL-1240685 has a very promising result that is worthy of further investigation in the treatment of Alzheimer's disease. It is worth mentioning that Zoladex and CHEMBL-1240685 are peptide compounds which, according to the *in silico* investigation, have shown to be possible compounds that could be used for the treatment of Alzheimer's disease; the side effect of Zoladex has been reported to cause temporary memory loss (Cancer Research UK [51]) in women and has shown that even though it is an FDA-approved drug, it will not be suggested as a possible drug molecule for the treatment of Alzheimer's disease. Finally, CHEMBL-1240685, which is a lipase inhibitor, is a new drug candidate that could be investigated further. Our focus is to now carry out a bioassay of CHEMBL-ID 1240685 as AChE inhibitors. This research has shown that *in silico* drug repurposing studies could be useful in the development of new drug candidates for the treatment of Alzheimer's disease.

### 4.1. Drug Repurposing Drawback

Drug repurposing might be an easy approach to shortening the time it takes to take a drug molecule from the laboratory to the market. It has been argued that pharmaceutical companies might not want to change the trademark of the drug from its original use [61]. This drawback might be addressed if companies collaborate [62]. Another drawback is a possible failure that has financial implications on the funder, and a typical example was the failure recorded in an attempt to repurpose bevacizumab for the treatment of other cancer diseases [63]. Despite these, drawbacks and others are described by Low et al. [50]. Drug repurposing remains a viable project to embark upon if the stakeholders (government, pharmaceutical companies, National Health Service, and academics) are intentional in urgently addressing the issue of lack of drugs to treat some diseases like Alzheimer's, and drug repurposing was very useful during COVID-19 outbreak.

### 4.2. Future Work

In this work, we have been able to show that CHEMBL-1240685 is a possible hit compound that can be investigated further for the treatment of Alzheimer's disease; our next step is to synthesise the compound, carry out *in vivo*, *in vitro* tests, pharmacokinetics, and pharmacodynamics evaluation of the compound.

## Figures and Tables

**Table 1:** CHEMBL-1240685, a peptide obtained from the ChEMBL database, showed a superior binding property during the molecular docking of experiments because it has a high binding affinity of -12.1467, and this was followed by Zoladex with the binding affinity of -11.2118.

Compound	Number of interactions	Types of interactions		*S* value (binding affinity)
		Hydrogen bond interaction	Hydrophobic interaction	
Donepezil	2		6-ring PHE 331 (H-pi) 6-ring TRP 279 (pi-pi)	-7.165

Galantamine	3	GLU-199 (H-donor) GLY 118 (H-acceptor)	6-ring PHE 331 (H-pi)	-6.3997

Zoladex	4	TYR-70 (H-donor) ASP72 (H-donor)	TRP 279 (pi-pi) LEU 282 (pi-H)	-11.2118

CHEMBL-1240685	8	SER 286 (H-donor) GLY-117 ASP 285 (H-donor) ASN-85 (H-donor) SER 286 (H-acceptor); ASP72 (H = donor)	Two TRP-279 (H-pi), PHE-330 (H-pi), TYR 334 (H-pi)	-12.1467

**Table 2:** Druglikeness profile of compounds investigated.

S/no.	Initial of compound	MW g/mol	No. of rotatable bond	H-acceptor	H-donor	LogP	LogS	BBB	GI permeability
1	Donepezil	379.49	6	4	0	4.91	-4.81	Yes	High
2	Galantamine	287.35	1	4	1	2.03	-2.93	Yes	High
3	Zoladex	970.08	34	15	12	-2.11	1.47	No	Low
4	CHEMBL-1240685	1269.41	43	16	17	1.36	-4.06	No	Low

**Table 3:** Toxicity results of the four compounds obtained from ProTox-II online tools.

S/no.	Compound	Mutagenicity	Hepatoxicity	Carcinogenicity	Immunotoxicity	Cytotoxicity	Predicted LD_50_ (mg/kg)	Predicted toxicity
1	Donepezil	Inactive	Inactive	Inactive	Active	Moderately active	505	4
2	Galantamine	Inactive	Inactive	Moderately inactive	Active	Moderately active	85	3
3	Zoladex	Moderately inactive	Inactive	Moderately inactive	Inactive	Moderately inactive	2400	5
4	CHEMBL-1240685	Moderately inactive	Inactive	Moderately inactive	Inactive	Inactive	3000	5

## Data Availability

All data are in the manuscript.
